# Tuberculosis in infants: a retrospective study in China

**DOI:** 10.1186/s40064-016-2184-7

**Published:** 2016-04-27

**Authors:** Ruo-Lin Li, Jun-Li Wang, Xin-Feng Wang, Mao-Shui Wang

**Affiliations:** Department of Medicine Research, First Affiliated Hospital of Guangxi Medical University, Nanning, Guangxi People’s Republic of China; Center of Clinical Laboratory, Affiliated Hospital of Youjiang Medical College for Nationalities, Baise, Guangxi People’s Republic of China; Department of Lab Medicine, Shandong Provincial Chest Hospital, 46# Lishan Road, Jinan City, 250013 People’s Republic of China

**Keywords:** Tuberculosis, Infant, China, Bacille Calmette–Guerin

## Abstract

To describe the demographics, clinical characteristics and microbiologic findings of infant (≤2 years old) tuberculosis (TB) in a high TB burden country. Between Feb, 2007 and Jun, 2015, 115 TB infants who admitted to our hospital were enrolled in the study. Their clinicopathological characteristics were reviewed and analyzed. The mean age was 10.1 ± 7.4 (SD) months, and 84 of 115 infants (73.0 %) were males. 23 patients (20.0 %) had isolated pulmonary TB, 18 patients (15.7 %) had pulmonary and extrapulmonary TB (EPTB), the remaining 74 patients (64.4 %) had exclusively EPTB. The most common site of EPTB was lymph node (n = 61), 54 cases were left axillary lymph node involvement. 49 of 51 patients (96.1 %) were validated by pathological examination, 5 of 57 patients (8.8 %) were positive on acid fast bacilli smear, and 27 of 103 patients (26.2 %) were confirmed by mycobacterial culture. 29 of 59 patients (49.2 %) were PPD positive, 14 of 30 patients (46.7 %) were T-SPOT.TB positive. The most common complaints of patients were lymph node swelling (53.0 %), fever (36.5 %), cough (28.7 %) and dyspnea (10.4 %). There was significant difference in the time before hospital admission among different types of tuberculosis (P < 0.01), fever was also a factor influencing the time (P < 0.05). In infants, the sensitivities of routine TB tests were low and emphasize the need for improved diagnostics; EPTB was more common than pulmonary TB, tuberculous lymphadenitis constituted a high proportion of EPTB; there appears to be an association between the incidence of axillary lymph node TB and BCG vaccination among infants in China.

## Background

Tuberculosis (TB) is a top infectious disease killer. In 2014, 9.6 million people fell ill with TB and 1.5 million died from the disease worldwide (Taghizade Moghaddam et al. 2016). China ranks second among the 22 high burden countries for TB. According to the fifth national TB surveillance in 2010, 1.3 million new cases of TB were estimated to occur each year, accounting for 14.3 % of incidental TB globally (Zhang et al. 2014). The prevalence of TB in eastern provinces including Shandong was 66 cases (95 % CI 52–84) per 100,000 population (Wang et al. 2014a). Therefore, TB remains a serious public health problem.

Because of limited contribution to TB transmission, childhood TB is often not considered a priority by national TB control programs. The long standing neglect of childhood TB has recently been addressed. WHO estimated that in 2012 there were 530,000 TB cases among children and 74,000 deaths among HIV-negative children (Starke 2014). In 2013, the Roadmap for childhood TB was launched, and the goal of achieving zero TB deaths in children was also adopted (Assefa et al. 2015). Unfortunately, there are several challenges facing childhood TB control: (1) TB in children is often missed or overlooked due to non-specific symptoms and difficulties in diagnosis, such as obtaining sputum from young children; (2) Due to the paucibacillary nature, the bacteriological tests have low sensitivity in detection of children TB; (3) Infants and young children are at increased risk of developing severe disseminated disease associated with high mortality, such as tuberculous meningitis or miliary TB (Rahman et al. 2012; Shingadia and Novelli 2003).

Bacille Calmette–Guerin (BCG) vaccine protects against disseminated TB in young children, and significantly reduces the risk of TB by 50 % (Trunz et al. 2006; Rodrigues et al. 1993; Colditz et al. 1994). However, BCG vaccination provides incomplete protection against TB in infants. Therefore, it’s worthwhile to assess clinical spectrum of infant TB to make a prompt diagnosis and effective treatment. In the retrospective study, we aimed to analyze 115 consecutive TB infants (≤2 years old) hospitalized during a 8-year period in our center, the demographics, clinical characteristics and microbiologic findings were discussed.

## Results

The mean age was 10.1 ± 7.4 (SD) months, and 84 of 115 infants (73.0 %) were males. 73 infants accepting HIV testing were all HIV-negative. 113 cases were BCG-vaccinated, two were unvaccinated. 23 of patients (20.0 %) had isolated pulmonary TB, 18 patients (15.7 %) had pulmonary and extrapulmonary TB, the remaining 74 cases (64.4 %) had exclusively extrapulmonary TB. The site of extrapulmonary TB included lymph node (n = 61), meningitis (n = 22), osteoarticular (n = 7), pleural (n = 2), middle ear (n = 1), peritoneal (n = 1) and genitourinary (n = 1). Among the tuberculous lymphadenitis patients, 53 cases were only diagnosed as tuberculous lymphadenitis (1 cervical and 52 left axillary lymph node involvement), 8 cases (2 left axillary, 1 lung, 1 mediastinal and 1 groin lymph nodes involvement) were co-existing other sites of TB. Miliary TB accounted for 17.4 % of all cases of TB and 31.5 % of TB cases without left axillary lymph node involvement. The contact source was identified in 15 cases (13.0 %), 93.3 % were members of the immediate family. Table 1 showed the clinicopathological characteristics of infant tuberculosis.

**Table 1:** Clinicopathological characteristics of infant tuberculosis(≤2 years old)

	Extrapulmonary TB	Pulmonary and extrapulmonary TB	Pulmonary TB	Overall
	n (%)	n (%)	n (%)	n (%)
Age (months)	10.6 ± 7.4	12.3 ± 7.7	7.0 ± 6.5	10.1 ± 7.4
Number	74	18	23	115
Male	57 (77.0 %)	14 (77.8 %)	13 (56.5 %)	84 (73.0 %)
HIV	0	0	0	0
BCG	73 (98.6 %)	18 (100 %)	22 (95.7 %)	113 (98.3 %)
Contact history	4 (5.4 %)	5 (27.8 %)	6 (26.1 %)	15 (13.0 %)
Miliary tuberculosis	0	8 (44.4 %)	12 (52.2 %)	20 (17.4 %)
Tuberculous meningitis	10 (13.5 %)	12 (66.7 %)	0	22 (19.1 %)
Pathological examination	43 (95.6 %)	2 (100 %)	4 (100 %)	49 (96.1 %)
Acid fast bacilli smear	5 (18.5 %)	0	0	5 (8.8 %)
Culture	15 (23.1 %)	6 (33.3 %)	6 (30.0 %)	27 (26.2 %)
PPD	24 (53.3 %)	3 (37.5 %)	2 (33.3 %)	29 (49.2 %)
T-SPOT.TB	7 (35.0 %)	5 (100 %)	2 (40.0 %)	14 (46.7 %)

*TB* tuberculosis, *HIV* human immunodeficiency virus, *BCG* Bacille Calmette–Guerin, *PPD* purified protein derivative

66 TB infants were confirmed by pathological examination or microbiological tests. 49 of 51 patients (96.1 %) were validated by pathological examination, 5 of 57 patients (8.8 %) were positive on acid fast bacilli smear, and 27 of 103 patients (26.2 %) were confirmed by the isolation of M.TB from bronchoalveolar lavage (n = 7), cerebrospinal fluid (n = 4), stool (n = 1) and tissues (n = 5). 29 of 59 patients (49.2 %) were PPD positive, 14 of 30 patients (46.7 %) were T-SPOT.TB positive.

The most common complaints of patients were lymph node swelling (53.0 %), fever (36.5 %), cough (28.7 %) and dyspnea (10.4 %). Among cases without isolated tuberculous lymphadenitis, fever (58.1 %) was the most common symptom, followed by cough (51.6 %) and dyspnea (19.4 %). Of the tuberculous meningitis cases, vomiting was found in 40.9 %, seizures occur in 31.8 %, coma in 22.7 %. Among the 54 patients with axillary lymph node TB, swollen lymph nodes of patients were all on the left and 13 patients with ulceration, the patients were all treated with anti-TB chemotherapy, 45 patients underwent surgical excision of their lymph nodes, and all of them showed a complete response to the combined treatment.

The time before hospital admission reflecting diagnostic delay was evaluated. There was significant difference in the time before hospital admission among different types of TB (P < 0.01). The average time was 75.5 ± 106.5 days for all patients, 38.9 ± 40.8 days for pulmonary TB, 91.8 ± 109.0 days for tuberculous lymphadenitis, 24.7 ± 14.9 days for tuberculous meningitis, 21.6 ± 19.0 days for pulmonary + extrapulmonary TB. Further analysis showed that fever was also a factor influencing the time (P < 0.05).

## Discussion

Unfortunately, until now there has not been an ideal diagnostic method in detection of childhood TB. In addition to this, TB children also face huge challenges: drug resistance, HIV infection, out of TB control program. In a Chinese study, data showed that 54 % of TB children had extrapulmonary TB, and a high percentage of extrapulmonary or severe TB was found in infants (Wu et al. 2012). Therefore, the retrospective study was conducted aiming to describe the detailed characteristics of infant TB. To our best of knowledge, this was the first report focusing on the clinical spectrum of infant TB.

Usually pulmonary TB is the first form of TB (Eurosurveillance Editorial Team 2013), cervical lymph nodes often constitute the most common site of involvement in tuberculous lymphadenitis while axillary nodes affected in a minority (Dandapat et al. 1990). But our study found that tuberculous lymphadenitis was the most common form of infant TB, followed by pulmonary TB and tuberculous meningitis, and the majority of cases were axillary lymph node involvement. Interestingly, all axillary lymph nodes located at left side. Therefore, the disease may be induced by BCG vaccination, because the left upper arm is the recommended site of BCG vaccination in China (Samuel et al. 2007). Meanwhile, a high percentage (83.3 %) of these patients who didn’t benefit from antituberculous therapy underwent surgical excision of their lymph nodes. The phenomenon may result from BCG increased resistance to anti-TB drugs (Kolibab et al. 2011; Ritz et al. 2009). Our data implied that, when BCG is given intradermally, adverse effects including enlargement of left axillary lymph node should be taken into account.

The diagnosis of TB disease in children is often more difficult than in adults because of non-specific signs and symptoms of TB and the presence of fewer mycobacteria, which results in fewer positive bacteriological tests (Nelson et al. 2004). Recently, a retrospective study assessing the 2012 NIH consensus criteria for standardized diagnostic categories of pulmonary tuberculosis in children showed that, there was necessary to perform further revision of the NIH definitions, because the entry criteria was too restrictive (Zar et al. 2015). This study implied children showed fewer symptoms than current knowledge indicated. Usually fever, cough and weight loss were the three most common symptoms for childhood pulmonary TB (González Saldaña et al. 2014). For childhood pulmonary TB, the incidence rates of fever and cough in the study were more likely to decrease than in several other studies (González Saldaña et al. 2014; Blount et al. 2014; Cruz et al. 2013; Shrestha et al. 2011), and nobody complained about weight loss. Fever was found to influence the time before hospital admission, it can be used to predictive of a short patient delay (Leutscher et al. 2012).

PPD was positive in 49.2 %, T-SPOT.TB in 46.7 %, while AFB and culture confirmation rates were low in our study, consistent with most other studies (González Saldaña et al. 2014; Perez-Velez and Marais 2012; Nelson et al. 2004). It looks like to be a high agreement between PPD and T-SPOT.TB. But PPD weren’t always performed in parallel with T-SPOT.TB, this should be taken into account. In China, T-SPOT.TB had a moderate sensitivity in diagnosis of childhood TB (Wang et al. 2014b). Fortunately, in recent studies, several assays have been evaluated and appear promising for detection of childhood TB, such as IP-10 (Holm et al. 2014; Latorre et al. 2014), urine lipoarabinomannan assays (Kroidl et al. 2015), Xpert MTB/RIF assay (Singh et al. 2015; Zar et al. 2013).

The study had several limitations. Although consecutive patients were enrolled, the retrospective nature may raise some questions concerning the representative nature of the cases. Since the treatment of infant patients was experience-dependent, the regimes of chemotherapy weren’t uniform and no further comparison was carried. All axillary lymph node TB was on the left side neighboring the recommended site of BCG vaccination. However, there was no direct proof that axillary lymph node TB was induced by BCG.

## Conclusions

We describe the demographics, clinical characteristics and microbiologic findings among TB infants admitted to a referral hospital in China. To sum up, we concluded that, (1) in infants, extrapulmonary TB was more common than pulmonary TB, tuberculous lymphadenitis was diagnosed in the majority of extrapulmonary TB patients; (2) unfortunately, PPD and T-SPOT.TB positivity which used to determine whether a patient is infected with M. TB were moderate in screening active TB in infants. Due to the paucibacillary nature of the disease in children, AFB and culture confirmation rates were low; (3) the incidence rates of common symptoms (such as fever, cough) in infants were more likely to be fewer than in other aged children; (4) there appears to be an association between the incidence of axillary lymph node TB and BCG vaccination among infants in China. When BCG is given intradermally, adverse effects including enlargement of left axillary lymph node should be taken into account.

## Methods

The study was conducted at Department of Lab Medicine, Shandong Provincial Chest Hospital and approved by the Ethics Committee of the Institute. Because of the retrospective nature, written consent was waived.

Between Feb, 2007 and Jun, 2015, 115 TB infants who admitted to our hospital were enrolled in the study. Their clinicopathological characteristics were reviewed and analyzed. TB infants were diagnosed based on TB contact, clinical symptoms (such as fever, cough and a response to anti-TB therapy), chest X-ray examination, PPD skin test, pathological examination and routine TB assays (such as mycobacterial culture, acid fast bacilli (AFB) smear, molecular tests, adenosine deaminase, serodiagnosis, T-SPOT.TB and erythrocyte sedimentation rate).

Statistical analysis was carried out using SPSS 17.0 software. Data were expressed as mean ± standard deviation (SD). Comparisons of data between different groups were performed using Kruskal–Wallis or Mann–Whitney tests. P < 0.05 was considered as statistically significant.

## Abbreviations

TB: tuberculosis
SD: standard deviation
EPTB: extrapulmonary tuberculosis
PPD: purified protein derivative
BCG: Bacille Calmette–Guerin
AFB: acid fast bacilli
ESR: erythrocyte sedimentation rate
